# Developing a Medical Education Framework for Migrant and Refugee Mental Health in Switzerland

**DOI:** 10.3389/ijph.2025.1608047

**Published:** 2025-04-22

**Authors:** Andrina Klöti, Fayez Abdulrazeq, Amina Trevisan, Tobias Eichinger, Fana Asefaw, Nikola Biller-Andorno, Julian W. März

**Affiliations:** ^1^ Institute of Biomedical Ethics and History of Medicine, Faculty of Medicine, University of Zurich, Zurich, Switzerland; ^2^ Prosalute, Basel, Switzerland

**Keywords:** medical education, curriculum development, medical education framework, refugee and migrant health, mental health of refugees and migrants

## Abstract

**Objectives:**

This study aimed to develop a medical education framework for migrant and refugee mental health in Switzerland, given substantial mental health challenges displaced people face along the phases of migration.

**Methods:**

Through informants from Swiss medical faculties, we assessed the existence of a comprehensive curriculum in migrant and refugee mental health. The curriculum was drafted based on a selective literature analysis, then reviewed, and verified by a committee of 17 experts in mental health of refugees and migrants. Their inputs have been used towards the development of the final framework.

**Results:**

No relevant comprehensive education framework existed, however, most medical schools provided related teaching. The literature analysis identified migration-related risk factors, social determinants and challenges when providing mental health to this population. Teaching methods included lectures, reflection rounds in small groups and practical experiences. The committee consisting of students, physicians and academics suggested a high level of approval for our initiative.

**Conclusion:**

Our method followed a pragmatic approach to suggest an expert-approved curriculum. Due to its comprehensiveness, only parts of it may be adopted into already existing study programs.

## Introduction

Given substantial mental health challenges displaced populations face before, during, and after migration, healthcare professionals must be adequately trained to provide effective care.

According to the International Organisation for Migration, there are 281 million international migrants globally, making up 3.6% of the world’s population [1, 2]. The number of forcibly displaced people worldwide is estimated at 108.4 million, with 40% under the age of 18 [3]. In Switzerland, over 2 million permanent residents are non-Swiss nationals, representing more than a quarter of the population [4]. In 2022, 40% of the population was considered to have a migration history, meaning they have migrated themselves or are part of the second generation. Currently, about 130,000 people in Switzerland are in the asylum process, mainly from Afghanistan, Eritrea, Syria, Turkey, and Ukraine, of which around 65,000 are holding temporary protection status [5].

Migrant and refugee populations are diverse, with varying countries of origin, residency status, socioeconomic backgrounds, and reasons for migration. They face multiple mental health risks across different stages, including pre-migration, movement, arrival, and sometimes return [6, 7]. Key risk factors include exposure to trauma, unfamiliarity with the health system, post-migration stress, racism, limited job access, unemployment, insecure residency, poor housing, and socio-economic challenges [8–17]. Studies have shown higher psychiatric morbidity in these groups, with asylum seekers in Switzerland often underdiagnosed and untreated and consulting healthcare professionals less than non-migrants [17–22].

Providing mental healthcare to refugees and migrants presents challenges such as language barriers, limited translation services, unfamiliarity with the local system, intersecting stigma, and cultural influences on illness behaviour, diagnosis, coping, and treatment [7, 10, 23–25]. Psychotherapy may be unfamiliar to certain people or stigmatised in some cultures, leading individuals to seek non-psychiatric services. Additionally, a lack of cultural diversity and transcultural competence, along with implicit bias and (latently) discriminating perceptions among healthcare providers, further complicates care [17, 26]. Findings from the U.S. indicate that the majority of medical students feel inadequately prepared to provide culturally sensitive care to migrants and refugees [27]. Future physicians should therefore be well-prepared to work with migrants and refugees, given the increasing need to address the health challenges faced by displaced people, and identify effective interventions [28].

This study aims to develop a basis for a curriculum to empower future physicians providing care to migrant and refugee populations in Switzerland with a focus on mental health. Our objectives include describing learning methods, identifying key content with literature references, outlining learning objectives, and exploring content delivery methods.

## Methods

### Phase 1: Identification of Existing Curricular Content

Based on our initial research, which involved reviewing university websites, we reached out to key informants via email. These informants were medical students at Swiss medical faculties in November 2022, recruited through the Swiss Study Foundation network. We chose to approach students to gain insight into their experiences with migrant and refugee mental health education at their institutions and to assess their general sense of preparedness in this area for their future work as physicians. Specifically, we sought information on the existence of a comprehensive curriculum in migrant and refugee mental health and requested details on related current course content.

### Phase 2: Literature Analysis

The literature analysis aimed to identify curricula and teaching content addressing migrant and refugee mental health in medical education. As found in phase one, no comprehensive curriculum existed in Switzerland, so we conducted a selective literature analysis approach. In addition to focussing on content, we also examined teaching and evaluation methods as well as learning objectives. We focused particularly on content which was relevant for addressing healthcare needs for migrants and refugees in Switzerland. We utilised the database PubMed, consulted the webpages of the Swiss government (Federal office of public health, State Secretariat for Migration) and of organisations such as the World Health Organisation (WHO) and the United Nations High Commissioner for Refugees (UNHCR). We extended our research on local organisations engaged in migrant and refugee mental health. We employed as search blocks migrants and refugees, medical education, and mental health. Articles in English, German and French were included.

### Phase 3: Developing a Migrant and Refugee Mental Health Framework

Based on the literature analysis, a framework for migrant and refugee mental health was drafted. Relevant topics, teaching activities, and opportunities for hands-on learning and evaluation were identified. The contents were grouped in themed sections by the lead author, suggesting teaching and evaluation methods, and practical experiences with reference to the relevant literature. Predominant mental health conditions and effective healthcare delivery strategies were highlighted, along with insights into social determinants of health and healthcare provision in Switzerland. After reviewing the draft, another author contributed practical expertise from working with migrants and refugees in a psychiatric setting, and the other authors reviewed and verified the content.

### Phase 4: Experts Review and Consultation

Between October 2023 and February 2024, our curriculum was reviewed by a diverse group of experts. We recruited medical students, (resident) physicians (mainly in internal medicine and psychiatry), and experts in migrant and refugee mental health with academic and/or professional and/or teaching experience as well as a background in medical education. The voluntary review aimed to include experts from different linguistic regions in Switzerland to enhance accuracy. Resident physicians were selected from the authors’ professional networks, medical students were recruited through the Swiss Study Foundation. Experts with established academic and/or practical and/or teaching expertise in the field—primarily focusing on medical professionals, though not limited to them—were identified through literature and online research. The experts were invited via email and asked to assess the curriculum’s relevance for teaching migrant and refugee mental health in Swiss medical schools and provide feedback by adding comments directly or submitting a separate document. Reviewed versions were returned via email.

#### Description of Experts

Out of 28 potential experts invited by email, 17 responded (a 61% response rate). Of these, 65% (11/17) identified as female and 35% (6/17) as male. The group included 12 medical graduates, three medical students, one expert with a Nursing Science background, and one from Sociology and Social Anthropology with migrant mental health research experience. Among the medical graduates, half (6/12) specialised or aimed to specialise in general internal medicine, 25% (3/12) in Psychiatry and Psychotherapy, and the remainder in Public Health, Neurology, or Child and Adolescent Psychiatry and Psychotherapy. In total, 65% (11/17) had current or past working activity with migrant and refugee health–seven with a focus on mental health–and 59% (10/17) shared current or past related research activity. Regarding personal migration experience, 52% (9/17) reported none, 24% (4/17) reported to have so, and 24% (4/17) were unsure. Those uncertain questioned whether factors like working as a French citizen in French-speaking Switzerland, as a German citizen in German-speaking Switzerland, being a second-generation migrant, or having lived in multiple countries qualified as migration experience. Finally, 12 participants were from the German-speaking region and 5 from the French-speaking region of Switzerland.

### Phase 5: Finalisation of the Framework

The feedback was analysed focussing on consensus, diverging views, suggested extensions of the curriculum, and prioritisation of certain content. Suggestions were incorporated into the curriculum and proposed references and literature by the experts have been consulted and, if suitable, integrated. The final version of the framework has been reviewed and agreed upon by all authors.

## Results

### Phase 1: Identification of Existing Curricular Content

In 2022, a total number of six medical students from Swiss faculties (Zurich, Basel, Bern, Geneva, Lausanne, St. Gallen) were consulted via email about existing curricula and their experiences with migrant and refugee mental health education at their institutions. No comprehensive curriculum for migrant and refugee mental health was identified. While all faculties offered psychiatry courses, none specifically focused on this topic. Migrant health was covered across faculties but primarily addressed infectious disease screening and primary care. All universities integrated content on migrants, vulnerable populations, and transcultural care. In one university (Bern), optional additional seminars on migration medicine and the healthcare of refugees were offered. Teaching methods included lectures, seminars, and practical research projects.

### Phase 2: Literature Analysis

Based on our literature and online research, we identified 90 records for developing our framework. The records comprised journal articles, books, and documents that discuss pertinent teaching subjects concerning migrant mental health, demographics, human rights, social determinants of health, provision of healthcare to migrant and refugee populations, trauma-related aspects, communication, medical humanities, and the role of networks. Additionally, the research encompassed an exploration of content delivery methods, as well as evaluation approaches.

### Phase 3: Developing a Migrant and Refugee Mental Health Framework

The topics identified within phases 1 and 2 allowed us to determine key teaching content, learning objectives, teaching methods, and evaluation approaches. The relevant content was structured into three sections: a theoretical section, a practical component, and an evaluation phase. The topics were assigned to lessons with the appropriate teaching method, time frame, and supporting literature.

The theoretical part provided an introduction encompassing the relevance of the topic, definitions of migrants and refugees, epidemiological data, and the legal framework of the Swiss asylum procedure, including residence statuses and their healthcare implications. It also covered human rights, social determinants of health, health equity, mental health assessment in migrant populations, reasons for and consequences of migration, forced displacement, risk factors at different migration stages, and barriers to healthcare access. A dedicated section encouraged reflection on bias and the lack of diversity among healthcare providers, while others focused on communication, trauma, and the role of networks.

The practical section included hands-on experiences such as working with community organisations, communication training with medical interpreters, and workshops with standardised patients. The third, evaluation phase, proposed a written reflection, medical history-taking exercises, and cultural competence assessments using standardised patient scenarios.

### Phase 4: Experts Review and Consultation

All experts strongly supported our initiative. Some medical professionals recommended expanding the curriculum beyond mental health to include topics such as infectious diseases and vaccination. Several experts suggested integrating the practical and the theoretical parts, while many contributed additional details and referenced local initiatives. The sections on humanities, clinical encounters, social determinants of health, and communication–particularly with practical experiences–were seen as highly relevant. Most experts favoured workshops, role-plays, and personal storytelling for content delivery. Primarily medical students emphasised the need for positive narratives in migrant and refugee mental health. Some experts questioned the curriculums’ scope given a limited lesson time or noted potential overlaps with existing medical coursework at faculties.

### Phase 5: Finalisation of the Framework

We adapted the curriculum based on expert feedback. The suggestion to combine the practical and theoretical parts led to the consolidation of the original three sections (theory, practice, and evaluation) into nine thematic blocks, with a separate (tenth) evaluation block. The first block introduces the topic, definitions, key statistics, and legal aspects of residence status in Switzerland. The subsequent blocks cover human rights, social determinants of health, migrant and refugee mental health, trauma, mental health services, communication, clinical encounter and medical humanities, and networks.

The suggestion to promote self-reflection was met by including sessions with a focus on own bias and attitudes, and own motivation for working in healthcare, as well as group workshops addressing prejudice, discrimination, and stigma. To foster positive narratives, we included sharing sessions involving students (e.g., reporting from their clinical rotations), refugees, and migrants, as suggested by experts. We also expanded the list of network and community partners, including those offering volunteer opportunities, translation services, and specialised care.

The recommendation to include non-mental health topics was not adopted due to the project’s focus on mental health as a particular important area. While experts raised concerns about time constraints and overlap with existing content, we left the time allocation flexible for implementation. The comprehensive framework can be found as a Supplementary File.

## Discussion

Considering the existing gaps in mental healthcare for migrants and refugees, and the growing migrant population in Switzerland, it is essential that future physicians develop a deep understanding of the mental health challenges these groups face. Our initiative was well-received by experts, highlighting the topic’s importance and the openness for implementation in practice. Studies show that medical students who complete refugee health programs often report shifts in their attitudes and beliefs or show improved confidence [27, 29–31]. Practical training might help students understand the interplay of cultural, social, and legal factors affecting health, encouraging critical reflection on their own biases, and expanding their perspectives.

Our approach presents a workable method to create evidence-based medical education content within a limited timeframe, balancing theory and practice. A diverse group of experts - encompassing medical students, resident physicians, and individuals with professional and/or research activity in the field–reviewed our curriculum, enhancing its comprehensiveness. We included general medicine physicians, acknowledging that mental health issues are not limited to psychiatric consultations, and a neurologist for their experience with migrant populations. The voluntary, high-response, and diverse nature of the expert group (including experts with non-medical backgrounds and with different experience levels) facilitated the development and review process. We did not impose strict criteria on the experts to ensure broad feedback.

Education frameworks for migrant and refugee health use different methodologies. In Canada, researchers developed a framework through a literature review and faculty interviews [32]. A 2024 UK curriculum was based on a systematic literature review [33]. Other programs focus on global health in family medicine, cross-cultural medical education and cultural competency, covering trauma-informed communication, mental healthcare, antiracism, advocacy, and religious considerations [34–40]. We draw on the Canadian approach but adapt it to Swiss contexts, considering differences in migrant demographics and socio-legal factors. Additionally, we used an expert-based approach rather than faculty interviews. Unlike the Canadian team´s focus on general migrant health, our curriculum prioritises mental health, addressing critical gaps and ensuring a more targeted scope.

This is the first comprehensive curriculum of its kind in Switzerland. To allow flexibility in implementation, we refrained from specifying a time frame. Acknowledging practical constraints such as limited time and resources, we recognise that medical faculties may adopt only parts of the curriculum, expand it, or integrate it into existing teaching. To illustrate a practical framework for implementing the curriculum within a limited time frame, please refer to Box 1. Key teaching content has been selected—for example, human rights are integrated into the introduction, trauma is addressed alongside providing care to the target population, and networks are incorporated into the clinical encounter section. The table outlines appropriate teaching methods and estimated hours for each section, considering potential time constraints in medical curricula and overlap with existing courses. It represents a flexible, shortened framework where relevant content and teaching methods can be adapted without strictly adhering to the full curriculum.

BOX 1 **Example of a Medical Education Framework for Migrant and Refugee Mental Health in Switzerland based on the comprehensive framework which can be found as a supplementary file including the relevant references. (Zürich, Switzerland. 2025).**

*Audience*: medical students in their last year of studies. *Amount of teaching hours*: 6 hours.

**Introduction**

*Content delivery method*: group lecture. *Time allocation*: 1 hour.

○ Emphasise the topic’s importance: explore service limitations, health equity, and differing perceptions of disease and health among patients and professionals, highlighting the need for specialised mental health skills for migrants and refugees.
○ Define *migrant*, *refugee*, *asylum seeker*, and *individuals with a migratory history* allowing for a broad perspective.
○ Examine global and Swiss-specific statistics on displaced populations, asylum seekers, and undocumented migrants.
○ Analyse healthcare access differences in Switzerland based on residence status.
○ Discuss (mental) health as a fundamental human right.

**Mental Health in Migrant and Refugee Populations and Social Determinants of Health**

*Content delivery method*: reflection rounds in moderated small groups. *Time allocation*: 1 hour.

○ Discuss factors influencing the mental health of migrants and refugees along the phases of migration. Explore post-migration living difficulties in depth.
○ Discuss the concept of social determinants of health and explore which factors may play a role in the mental health of refugees and migrants.

**Providing Mental Health Care to Migrant and Refugee Populations**

Content delivery method: group lecture. Time allocation: 1 hour.

○ Explore healthcare professionals' barriers, including but not limited to lack of cultural competence and sensibility, diversity, and language challenges.
○ Reflect on diverse views of (mental) health, disease, and mental health as a Western concept that may unfold in many ways globally.
○ Consider potential reluctance to seek care due to fears related to residency status and public authorities.
○ Define trauma and discuss post-traumatic stress disorder (PTSD), including risk factors, symptoms, and screening, while avoiding an oversimplified view of the refugee experience as a trauma-related issue.
○ Learn the principles of trauma-informed care.

**Communication**

*Content delivery method*: workshops and role plays with professional medical interpreters and standardised patients. *Time allocation*: 2 hours in small groups of around 6 students.

○ Perform a medical assessment with a standardised patient and a medical interpreter.

**Clinical Encounter and Networks**

*Content delivery method*: moderated small groups including testimonials by refugees/migrants and students. *Time allocation*: 1 hour.

○ Reflect on personal and systemic biases, racism, and (intersectional) discrimination, and explore ways to raise awareness, promote social accountability, and advocate as a medical professional.
○ Identify key local members of the network (e.g., family, social workers, communities, healthcare providers) and explore organisations for engagement and learning.

**Evaluation**

○ Prepare a written reflection (2,000 words) on what has been learned and skills gained with a focus on how students’ perspective has changed regarding healthcare for migrants and refugees.

While some experts suggested expanding the scope beyond mental health, we focused on mental health as we saw the need to raise medical students’ awareness regarding mental health of those populations as particularly important and underrepresented during medical studies. Expanding the curriculum further would increase its comprehensiveness, but may also lead to an overwhelming scope, making it difficult to maintain practical applicability within the constraints of medical education. However, our findings might not only be applicable when dealing with mental health issues in migrant and refugee care, which might lead to a more holistic approach to migrant and refugee health.

Defining *migrants* and *refugees* was challenging, as migration histories vary greatly, as do social determinants of health and disparities in access to care. We acknowledge that certain migrant groups might be part of a socioeconomically advantaged, medically well-supplied group of the population that is not the primary addressee of this curriculum. Not all experts could definitively answer whether they identified with being a migrant, reflecting the complexity of defining migrant populations. A more flexible definition may be necessary for providing comprehensive care.

### Limitations

A more detailed investigation into the curricula of all Swiss medical faculties was beyond the scope of this study. However, our primary aim was to determine whether comprehensive curricula in migrant and refugee mental health exist, rather than to provide an exhaustive analysis of all course offerings. Our research predominantly reflects the major medical faculties (Zurich, Bern, Basel, Geneva, Lausanne), while some smaller faculties that collaborate with these institutions (Lucerne, ETH, Lugano, Fribourg, Neuchâtel) are only partially represented. St. Gallen was included in our study. Additionally, our findings focus exclusively on undergraduate medical education and do not cover specialist training. This focus is justified, as a foundational understanding of migrant and refugee mental health is essential for all future physicians, regardless of their chosen specialisation.

Not all participants were designate experts in migrant health, such as some medical students and resident physicians without direct working and/or research experience with these populations. However, their perspectives hold significance given that the curriculum will be targeted at them and aims to enhance their understanding. We assumed that current students and junior doctors would provide insight into what content and which teaching delivery methods would be most relevant for them.

Additionally, while we briefly addressed racism in medicine as a relevant risk factor for mental health, we acknowledge that its impact deserves deeper exploration.

### Conclusion

In light of the mental health challenges faced by migrants and refugees, healthcare professionals must be trained to provide effective care. Our study addresses this gap by developing a medical education framework highlighting social determinants of health, human rights, epidemiology, and specific mental health risks. Though not empirical, it offers a practice-oriented, expert-driven approach, emphasising local network involvement. The framework provides medical faculties with essential teaching content, methods, practical experiences, and evaluation strategies to enhance self-reflection and improve care for migrants and refugees, particularly in the context of mental health in Switzerland. By positioning education as a key enabler for improving healthcare provision, the framework facilitates a system-level impact on migrant and refugee mental health.
