# Supplementary material for: Developing a Medical Education Framework for Migrant and Refugee Mental Health in Switzerland
Source: Int J Public Health. 2025 Apr 22;70:1608047. doi: 10.3389/ijph.2025.1608047 (PMC12053126; doi:10.3389/ijph.2025.1608047)
Supplement: Supplementary file 1 [file DataSheet1.PDF]

A Medical Education Framework for Migrant and Refugee Mental Health in Switzerland

Audience: medical students in their last 2 years of studies

Content delivered by professors, community physicians, guest speakers, specialised nurses, social workers, sociologists, medical anthropologist [1, 2] and other professionals with experience in mental health of migrants and refugees, ensuring diversity among speakers from various cultural backgrounds (including refugees and migrants) and regular reflection rounds in small groups throughout the course. E-learning / internet-based learning modules may be applicable to mainly theoretical content [3].

Introduction

- Content delivery method: group lecture or e-learning.
- Highlight the importance of the topic.
  - Show the current situation: available services often do not meet the linguistic, socioeconomic, and cultural needs of the population. Be aware that asylum seekers might be underdiagnosed and, as a result, receive inadequate treatment [4].
  - Learn about the concept of health equity [5].
  - Understand different perceptions and explanatory models of disease and health, both from the patients' and health care professionals' perspectives.
  - Recognise the need for specific skills and knowledge regarding mental health in migrant and refugee populations, including the importance of the relationship between health care professional and patient, establishing epistemic trust, transcultural communication, sensitivity, and cross-cultural competencies [6].
  - Remember the salutogenic approach and biopsychosocial conception of health: focusing on strengthening resources (vs. deficits), avoiding pathologizing, and acknowledging the importance of social determinants of health.
  - Reflect on one's own conception of humans and personal role as a healthcare provider.
  - Understand that the first medical encounter for asylum seekers often happens by specialised nurses for triage purposes, followed by primary health institutions or emergency departments (without specific training).
- Definitions
  - Define the terms *migrant*, *asylum seeker*, *refugee*, and *residents with a migration history*, including definitions by the International Organization for Migration (IOM) [7] and by the United Nations High Commissioner for Refugees (UNHCR) [8].
  - Explain different migration types, including internal vs. international migration and forced vs. planned migration, while allowing for a broader definition of people with a migratory history.
  - Provide personal stories from migrants and refugees reflecting on what being a migrant or refugee means to them and their lived experiences.
- Statistics
  - Examine global statistics on forcibly displaced people, asylum seekers, and undocumented migrants, with a specific focus on Switzerland. Include data on country of origin, host countries, demographics (such as gender and age, and particularly unaccompanied minor asylum seekers), and types of residency permits [9-13].
  - Provide an overview of permanent migrants in Switzerland, including their country of origin, demographic details, and residency permit types.
  - Discuss major migration routes, risks encountered along journey, pushbacks and detention practices.
- Legal bases on residence status in Switzerland
  - Review graphics on asylum procedure, including information on Dublin procedure, accelerated procedure, extended procedure, as well as the duration and accommodation in asylum centres [14].
  - Understand the differences in healthcare access based on residence status [15-18]. Permit B & C holders are eligible for compulsory health insurance, as are Permit N (Asylum-seekers), Permit F (provisionally admitted Asylum-seekers) and Permit S (people in need for protection, first introduced in March 2022 and currently valid only for individuals from

    Ukraine). Those with uncertain residence status, however, do not have the same entitlements.
  - Discuss uncertain residence status [19], including rejected asylum seekers entitled to *Nothilfe* (emergency relief) under Article 12 of the Federal Constitution of the Swiss Federation and those affected by a *Nichteintretensentscheid*. Explain the term *sans-papier* [12, 20, 21] and their access to healthcare in Switzerland [22].

Human Rights

- Content delivery method: group lectures or e-learning.
- Discuss Article 14 of the Universal Declaration of Human Rights: The right to seek and enjoy asylum.
- Discuss Article 25 of the Universal Declaration of Human Rights: the right to health, medical care, and an adequate standard of living.
  - Discuss how this might differ in reality, where access to healthcare is often limited, varies by country, and depends on insurance coverage. Residence in collective living quarters frequently face a lack of privacy and poor living conditions.
  - Discuss health and mental health as fundamental and inclusive human rights [23-25].
- Discuss Article 23 of the Universal Declaration of Human Rights: the right to work [26].
  - Holders of residence permits B, F, and S have unrestricted access to the labour market. However, employment opportunities may be limited due to language barriers, leading some to only find unpaid internships or remain unemployed.
  - Permit N holders (persons in ongoing asylum procedures) face restricted labour market access and are generally barred from working for the first 3-6 months.
  - Rejected asylum seekers and undocumented migrants do not have the right to work.
  - Explore how the lack of employment can negatively impact mental health, reduce self-esteem, lead to financial dependence (e.g., reliance on social welfare or a partner), and hinder social inclusion.

Social Determinants of Health

- Content delivery method: workshops and moderated small-group reflection rounds.
- Understand that differences in social, economic, and environmental circumstances may lead to health inequities.
- Explore social determinants of health such as income, financial stability, job security, healthcare access, gender, education, language, culture, racism, trauma, non-recognition of diplomas, (multiple) discrimination, power imbalances in binational relationships, lack of social support, exclusion, lack of appreciation, absence of belonging, social isolation, uncertainties regarding residency status, and fear of expulsion due to claiming of social benefits [5, 24, 27-31].
- Reflect in group discussions on which social determinants of health specifically impact refugees and migrants, and consider how these factors may influence their health outcomes.
- As a case study, discuss inequities during COVID-19 [32, 33], including issues like overcrowded collective housing, lack of masks and disinfectant, and lower vaccination rates due to insufficient information. Also, consider cantonal differences.
- Understand the interrelated nature of practical challenges (e.g. residence permits, inclusion, cultural diversity, language barriers, employment, housing) and mental health concerns (e.g. feelings of frustration, uncertainty and injustice) [34].
- Reflect on possible solutions to address health inequities in group discussions.

Mental Health in Migrant and Refugee Populations

- Content delivery method: small group lectures with reflection rounds.
- Discuss factors influencing the mental health of migrants and their families along the phases of migration [35].
  - Elaborate pre-, peri- and post-migration factors that may influence (mental) health, reflecting on the challenges involved.
  - Recognise the potential additional burden on asylum seekers and refugees from traumatic experiences in the country of origin, during migration, and throughout the asylum process.
- Be aware of differences between health systems, unfamiliarity with the local health system, and varying levels of health literacy [36, 37].

- Review social determinants of health, particularly in relation to mental health in migrant and refugee populations [24, 27-29, 38].
    - Discuss post-migration living difficulties, such as social isolation, language barriers, lack of social and professional participation, racism, poverty, distrust and possible fear of authorities or deportation, lack of stability and perspective, and unclear residence status including uncertainty about the duration of the asylum procedure [30, 39-48].
- Address mental health issues in migrants and refugees
    - Consider the rise of the global burden of disease due to mental disorders due to major demographic, environmental, economic, and socio-political transitions [24], and provide a demographic overview on migrants' and refugees' health status and healthcare access in Europe and in conflict-affected regions [30, 49-51].

Trauma

- Content delivery method: mixed group lectures by guest speakers with expertise in trauma care and individuals with lived experience.
- Define trauma based on the post-traumatic stress disorder (PTSD) definition by WHO ICD-11 02/2022[1].
- Discuss PTSD, including psychodynamics, risk and protective factors, screening questions, and symptoms. Value resilience, sub-threshold symptoms, reluctance to pathologise, and cultural variation in expressing and coping with trauma. Teach how to recognise symptoms and when to refer individuals to specialists.
- Explore different experiences along the migration journey, including arrival in the host country, human trafficking, genital mutilation, rape (to both sexes), and torture [52, 53].
- Discuss trauma and somatisation [54, 55], focusing on diffuse complaints that do not respond to somatic therapy, such as body intrusions, sleeping disorders due to nightmares or arousal/agitation, and the interest of body-mind therapies.
- Address intergenerational and sequential trauma, explaining how parents' post-traumatic stress and depressive symptoms may affect their children's psychosocial adaptability [56], also explain epigenetic changes.
- Consider comorbidities with PTSD, such as depression and substance abuse.
- Mind the danger of an over oversimplifying understanding of the refugee experience as a trauma-related issue [57].
- Explain psychoeducational aspects of trauma, providing information on PTSD in multiple languages [58].
- Introduce the principles of trauma-informed care, including as awareness, safety, trustworthiness, choice, collaboration, connection, strengths-based approaches, and skill building [59-61].
- Understand the association between psychological impairment in treatment-seeking traumatised individuals and poor inclusion, emphasising that the greater the mental impact, the worse the inclusion [62].

Providing Mental Health Services in Migrant and Refugee Populations

- Content delivery method: group workshops.
- Discuss the challenges and barriers in providing (mental) healthcare to refugees and migrants, including cultural and socio-cultural obstacle [63, 64].
- Reflect on diverse perceptions of health and disease, particularly mental illness, across different health systems. Address how "mental health" as a Western concept may unfold in many ways

[1] "Exposure to an event or situation (either short- or long-lasting) of an extremely threatening or horrific nature. Such events include, but are not limited to, directly experiencing natural or human-made disasters, combat, serious accidents, torture, sexual violence, terrorism, assault or acute life-threatening illness (e.g., a heart attack); witnessing the threatened or actual injury or death of others in a sudden, unexpected, or violent manner; and learning about the sudden, unexpected or violent death of a loved one."

globally, including differences in symptom presentation, disease models, and role understandings. Discuss the implications of stigma, potential rejection, and mistrust from the beginning.
- Consider potential reluctance to access healthcare services due to concerns about public authorities, particularly in cases involving uncertain residency status, such as fears of arrest or ineligibility for healthcare benefits.
- Adress barriers among healthcare professionals, including insufficient knowledge, cultural insensibility, paternalism, lack of diversity, lack of cultural competence and cultural humility, and potential misdiagnosis due to language barriers and varying understanding of mental illness [65]. Reflect on the crucial role of language in healthcare delivery.
- Acknowledge the challenges arising from limited availability, knowledge, and experience with translation services, including denial of treatment due to linguistic barriers, reliance on family members as interpreters, and high costs [66-73].
- Develop strategies to overcome these barriers, such as creating screening tools to help identify mental health challenges in migrant and refugee populations [74].

Communication

- Content delivery method: workshops and role-plays, and training with professional medical interpreters, standardised patients, or in interprofessional teams [2, 3, 75-78].
- Conduct a medical assessment at specialised medical centres or at family physicians' practices.
- Differentiate between verbal and non-verbal communication and recognise cultural nuances.
- Discuss barriers and facilitators in transcultural communication, focusing on culture-specific aspects, and sensitivity to religious differences, with potential support from social workers [79-81].

Clinical Encounter and Medical Humanities

- Content delivery method: workshops in groups, including sharing experiences and testimonials from students and refugees/migrants [1, 76], and introduce the concept of a human library.
- Explore mindful interaction and raise awareness to increase social accountability [82], integrating advocacy while ensuring culturally sensitive encounters that respect privacy. Reflect on cultural competence versus cultural humility.
- Reflect on personal and medical system biases, including prejudice, discrimination, stereotypes, and both explicit and implicit bias. Discuss equity vs. equality.
- Analyse stigma through a framework for assessment [83, 84], considering intersectional stigma [85] across mental health, migration, gender, sexual orientation, and economic situation. Explore how stigma acts as a barrier to mental health and its impact on migrant and refugee populations [86, 87].
- Reflect on personal motivations for working in healthcare, the local medical culture, and the experience of working within a transdisciplinary network.
- Consider the issues of racism [88], collecting data on race and ethnicity [89], and the role of racism as a catalyst for PTSD and related symptoms [90].
- Discuss the ethical conflict between providing medical care and service restrictions based on migratory status [91].
- Participate in a module on self-care, focusing on managing emotional overload among healthcare professionals.
- Explore the health needs of other vulnerable groups, such as socioeconomically disadvantaged individuals, homeless people, and sex workers. Reflect on multiple discrimination and intersectionality (e.g., migration, low educational level, psychological comorbidities, gender, and minors).

Networks

- Content delivery method: Reflect in small groups about the local network.
    - Reflect about the network's members (e.g., family, translators, family physicians, other health professionals, social workers, psychologists, faith-based/religious communities) [92, 93]. Consider their roles, such as destigmatising mental health.
- Introduction to non-exhaustive list of local organisations and opportunities for engagement as well as further learning:
    - Teaching method: presentation of organisations in group lecture.
    - National platform for sans-papiers health care providing information on contact points [13].

- Network of five specialized outpatient clinics in Switzerland for victims of torture and war, offering referrals and work shadowing opportunities: https://www.torturevictims.ch/ .
- Special facilities and treatment centres for migrant and refugee mental health, including transcultural consultations: for example https://www.appartenances.ch/ .
- Short interventions for mentally distressed asylum seekers: https://www.bag.admin.ch/bag/de/home/strategie-und-politik/nationale-gesundheitsstrategien/gesundheitliche-chancengleichheit/chancengleichheit-in-der-gesundheitsversorgung/gesundheit-asylbereich.html#-1640583780.
- Volunteering opportunities in Switzerland : https://www.fluechtlingshilfe.ch/aktiv-werden/freiwilligenprojekte.
- Free legal counselling for asylum seekers and temporarily admitted persons (organised by the canton).
- Victim counselling services and regional women's shelter (organised by the canton).
- Examples of translator services (by phone or on site) : https://www.inter-pret.ch/; https://www.stadt-zuerich.ch/medios.
- FIZ: Advocacy and Support for Migrant Women and Victims of Trafficking: https://www.fiz-info.ch/en/Welcome.
- Other non-medical non-governmental organisations such as: Solinetz, Sportegration (https://sportegration.ch/) , Paxion (https://www.paxion.ch/), Brückenbauer:innen (https://ncbi.ch/integration-von-gefluechteten/brueckenbauerinnen/) , SPIRIT (https://www.spirit-network.ch/), Prosalute (https://www.prosalute.ch/).
- Local ambulatory community care consultations (such as https://www.hug.ch/medecine-premier-recours/camsco).
- Further learning sources by the World Health Organisation [94, 95] and platforms such as Coursera and OpenWHO (https://www.coursera.org/; https://openwho.org/).

- Practical experience:
    - Gather information on the local network in your town, participate in working with a community organisation [77], and engage in volunteering activities.

Evaluation

- Prepare a written reflection on what has been learned and competences gained [77].
- Assess medical history taking and cultural competences in standardised patient situations.

References

1. Dussán KB, Galbraith EM, Grzybowski M, Vautaw BM, Murray L, Eagle KA. Effects of a refugee elective on medical student perceptions. BMC Med Educ. 2009;9:15.
2. Griswold K, Zayas LE, Kernan JB, Wagner CM. Cultural awareness through medical student and refugee patient encounters. J Immigr Minor Health. 2007;9(1):55-60.
3. Pottie K, Hostland S. Health advocacy for refugees: Medical student primer for competence in cultural matters and global health. Can Fam Physician. 2007;53(11):1923-6.
4. Maier T, Schmidt M, Mueller J. Mental health and healthcare utilization in adult asylum seekers. Swiss Med Wkly. 2010;140:w13110.
5. Health FFOoP. Health Equity 2023 [Available from: https://www.bag.admin.ch/bag/en/home/strategie-und-politik/nationale-gesundheitsstrategien/gesundheitliche-chancengleichheit.html.
6. Domenig D. Transkulturelle und transkategoriale Kompetenz. Lehrbuch zum Umgang mit Vielfalt, Verschiedenheit und Diversity für Pflege-, Gesundheits- und Sozialberufe: Hogrefe; 2021.
7. IOM IOfM. Who is a Migrant? IOM Definition of "Migrant" 2019 [Available from: https://www.iom.int/who-migrant-0.
8. UNHCR. Migrant Definition United Nations High Commissioner for Refugees2022 [Available from: https://emergency.unhcr.org/entry/44937/migrant-definition.
9. UNHCR. Refugee Statistics United Nations High Commissioner for Refugees 2022 [Available from: https://www.unhcr.org/refugee-statistics/.
10. SEM. State Secretariat for Migration - Foreign Population Statistics 2022 [Available from: https://www.sem.admin.ch/sem/de/home/publiservice/statistik/auslaenderstatistik.html.
11. SEM. State Secretariat for Migration - Asylstatistik 2022 [Available from: https://www.sem.admin.ch/sem/de/home/publiservice/statistik/asylstatistik.html.
12. FCM. The Federal Commission on Migration - Sans-Papier 2022 [Available from: https://www.ekm.admin.ch/ekm/de/home/zuwanderung---aufenthalt/sanspapiers.html.
13. sante-sans-papiers. plate-forme nationale pour les soins de santé aux sans-papiers 2016 [Available from: http://www.sante-sans-papiers.ch/DE/index.html.
14. SEM. State Secretariat for Migration - Procédure d'asile à partir de 2019 2019 [Available from: https://www.sem.admin.ch/dam/sem/fr/data/asyl/beschleunigung/grafik-asylverfahren.pdf.download.pdf/grafik-asylverfahren-f.pdf.
15. Fakhoury J, Burton-Jeangros C, Consoli L, Duvoisin A, Jackson Y. Association Between Residence Status Regularization and Access to Healthcare for Undocumented Migrants in Switzerland: A Panel Study. Front Public Health. 2022;10:832090.
16. Levesque JF, Harris MF, Russell G. Patient-centred access to health care: conceptualising access at the interface of health systems and populations. Int J Equity Health. 2013;12:18.
17. SEM. State Secretariat for Migration - Residence Permits for non-EU/EFTA nationals 2019 [Available from: https://www.sem.admin.ch/sem/en/home/themen/aufenthalt/nicht_eu_efta.html.
18. Katja N, Katharina L. Gesundheitswegweiser Schweiz. Federal Office of Public Health, Swiss Red Cross; 2017.
19. SFH. Schweizerische Flüchtlingshilfe - Aufenthaltsstatus 2022 [Available from: https://www.fluechtlingshilfe.ch/themen/asyl-in-der-schweiz/aufenthaltsstatus.
20. SEM. State Secretariat for Migration - Nothilfe 2019 [Available from: https://www.sem.admin.ch/sem/de/home/asyl/sozialhilfesubventionen/nothilfe.html.
21. Bundesrat. Gesamthafte Prüfung der Problematik der Sans-Papiers, Bericht des Bundesrats in Erfüllung des Postulats der Staatspolitischen Kommission des Nationalrats vom 12. April 2018 (18.2281). 2020.
22. FOPH. Healthcare provisions for undocumented migrants: Federal Office of Public Health; 2022 [Available from: https://www.bag.admin.ch/bag/en/home/strategie-und-politik/nationale-gesundheitsstrategien/gesundheitliche-chancengleichheit/chancengleichheit-in-der-gesundheitsversorgung/gesundheitsversorgung-der-sans-papiers.html.
23. WHO&OHCHR. World Health Organization & Office of the United Nations High Commissioner for Human Rights - Fact Sheet No. 31: The Right to Health. 2008.

24. Patel V, Saxena S, Lund C, Thornicroft G, Baingana F, Bolton P, et al. The Lancet Commission on global mental health and sustainable development. Lancet. 2018;392(10157):1553-98.
25. OHCHR. OHCHR and the right to health 2023 [Available from: https://www.ohchr.org/en/health.
26. Romy K, Leysinger V. Viele Geflüchtete finden in der Schweiz keine Arbeit 2023 [Available from: Viele Geflüchtete finden in der Schweiz keine Arbeit - SWI swissinfo.ch.
27. Marmot M, Allen JJ. Social determinants of health equity. Am J Public Health. 2014;104 Suppl 4:S517-9.
28. WHO. World Health Organization - Social determinants of health 2022 [Available from: https://www.who.int/health-topics/social-determinants-of-health#tab=tab_3.
29. Gruner D, Feinberg Y, Venables MJ, Shanza Hashmi S, Saad A, Archibald D, et al. An undergraduate medical education framework for refugee and migrant health: Curriculum development and conceptual approaches. BMC Med Educ. 2022;22(1):374.
30. Trevisan A. Depression und Biographie: Transcript; 2019.
31. WHO. Integrating the social determinants of health into workforce education and training2023.
32. Greenaway C, Hargreaves S, Barkati S, Coyle CM, Gobbi F, Veizis A, et al. COVID-19: Exposing and addressing health disparities among ethnic minorities and migrants. J Travel Med. 2020;27(7).
33. Alarcão V, Virgolino A, Stefanovska-Petkovska M, Neves J. Exploring the Effects of the COVID-19 Pandemic on Mental Health and Well-Being of Migrant Populations in Europe: An Equity-Focused Scoping Review. Behav Sci (Basel). 2022;12(10).
34. Kiselev N, Pfaltz M, Schick M, Bird M, Pernille H, Sijbrandij M, et al. Problems faced by Syrian refugees and asylum seekers in Switzerland. Swiss Med Wkly. 2020;150:w20381.
35. Migration-Data-Portal. Migration and Health 2021 [Available from: https://www.migrationdataportal.org/themes/migration-and-health.
36. Maillefer F, Bovet É, Jaton L, Bodenmann P, Ninane F. "Ma Santé, elle est toujours malade" : asile, santé et accès aux soins pour les requérants d'asile du canton de Vaud. Rev Med Suisse. 2021;17(734):754-7.
37. Medina P, Maia AC, Costa A. Health Literacy and Migrant Communities in Primary Health Care. Front Public Health. 2021;9:798222.
38. Marmot M, Bell R. Fair society, healthy lives. Public Health. 2012;126 Suppl 1:S4-S10.
39. Steel Z, Silove D, Brooks R, Momartin S, Alzuhairi B, Susljik I. Impact of immigration detention and temporary protection on the mental health of refugees. Br J Psychiatry. 2006;188:58-64.
40. Laban CJ, Gernaat HB, Komproe IH, van der Tweel I, De Jong JT. Postmigration living problems and common psychiatric disorders in Iraqi asylum seekers in the Netherlands. J Nerv Ment Dis. 2005;193(12):825-32.
41. Wicki B, Spiller TR, Schick M, Schnyder U, Bryant RA, Nickerson A, et al. A network analysis of postmigration living difficulties in refugees and asylum seekers. Eur J Psychotraumatol. 2021;12(1):1975941.
42. Bogic M, Njoku A, Priebe S. Long-term mental health of war-refugees: a systematic literature review. BMC Int Health Hum Rights. 2015;15:29.
43. Hainmueller J, Hangartner D, Lawrence D. When lives are put on hold: Lengthy asylum processes decrease employment among refugees. Sci Adv. 2016;2(8):e1600432.
44. Heeren M, Wittmann L, Ehlert U, Schnyder U, Maier T, Müller J. Psychopathology and resident status - comparing asylum seekers, refugees, illegal migrants, labor migrants, and residents. Compr Psychiatry. 2014;55(4):818-25.
45. Jackson Y, Courvoisier DS, Duvoisin A, Ferro-Luzzi G, Bodenmann P, Chauvin P, et al. Impact of legal status change on undocumented migrants' health and well-being (Parchemins): protocol of a 4-year, prospective, mixed-methods study. BMJ Open. 2019;9(5):e028336.
46. Fakhoury J, Burton-Jeangros C, Consoli L, Duvoisin A, Courvoisier D, Jackson Y. Mental health of undocumented migrants and migrants undergoing regularization in Switzerland: a cross-sectional study. BMC Psychiatry. 2021;21(1):175.

47. Fakhoury J, Burton-Jeangros C, Guessous I, Consoli L, Duvoisin A, Jackson Y. Self-rated health among undocumented and newly regularized migrants in Geneva: a cross-sectional study. BMC Public Health. 2021;21(1):1198.
48. Trevisan A. Depression und Biographie. Krankheitserfahrungen migrierter Frauen in der Schweiz.: Transcript; 2020.
49. Lebano A, Hamed S, Bradby H, Gil-Salmerón A, Durá-Ferrandis E, Garcés-Ferrer J, et al. Migrants' and refugees' health status and healthcare in Europe: a scoping literature review. BMC Public Health. 2020;20(1):1039.
50. Charlson F, van Ommeren M, Flaxman A, Cornett J, Whiteford H, Saxena S. New WHO prevalence estimates of mental disorders in conflict settings: a systematic review and meta-analysis. Lancet. 2019;394(10194):240-8.
51. Turrini G, Purgato M, Ballette F, Nosè M, Ostuzzi G, Barbui C. Common mental disorders in asylum seekers and refugees: umbrella review of prevalence and intervention studies. Int J Ment Health Syst. 2017;11:51.
52. Li SS, Liddell BJ, Nickerson A. The Relationship Between Post-Migration Stress and Psychological Disorders in Refugees and Asylum Seekers. Curr Psychiatry Rep. 2016;18(9):82.
53. Steel Z, Chey T, Silove D, Marnane C, Bryant RA, van Ommeren M. Association of torture and other potentially traumatic events with mental health outcomes among populations exposed to mass conflict and displacement: a systematic review and meta-analysis. JAMA. 2009;302(5):537-49.
54. Lanzara R, Scipioni M, Conti C. A Clinical-Psychological Perspective on Somatization Among Immigrants: A Systematic Review. Front Psychol. 2018;9:2792.
55. Morina N, Kuenburg A, Schnyder U, Bryant RA, Nickerson A, Schick M. The Association of Post-traumatic and Postmigration Stress with Pain and Other Somatic Symptoms: An Explorative Analysis in Traumatized Refugees and Asylum Seekers. Pain Med. 2018;19(1):50-9.
56. Asefaw F, Bombach C, Wöckel L. In der Schweiz lebende Minderjährige mit Fluchterfahrungen. Swiss Archive of Neurology, Psychiatry and Psychotherapy2018.
57. Varvin S. Unser Verhältnis zu Flüchtlingen: zwischen Mitleid und Entmenschlichung. Psyche. 2018;3(72. Jahrgang):194-215.
58. Hofer T. Schweizerisches Rotes Kreuz, Gesundheit und Integration - Wenn das vergessen nicht gelingt, Informationsbroschüre zur Posttraumatischen Belastungsstörung. 2022.
59. Dawson S, Bierce A, Feder G, Macleod J, Turner KM, Zammit S, et al. Trauma-informed approaches to primary and community mental health care: protocol for a mixed-methods systematic review. BMJ Open. 2021;11(2):e042112.
60. BC. Provincial Mental Health and Substance Use Planning Council - Trauma-Informed Practice Guide. 2013.
61. Maier T, Morina N, Schick M, Schnyder U. Trauma - Flucht - Asyl: Hogrefe; 2019.
62. Schick M, Zumwald A, Knöpfli B, Nickerson A, Bryant RA, Schnyder U, et al. Challenging future, challenging past: the relationship of social integration and psychological impairment in traumatized refugees. Eur J Psychotraumatol. 2016;7:28057.
63. Kiselev N, Pfaltz M, Haas F, Schick M, Kappen M, Sijbrandij M, et al. Structural and socio-cultural barriers to accessing mental healthcare among Syrian refugees and asylum seekers in Switzerland. Eur J Psychotraumatol. 2020;11(1):1717825.
64. Kiselev N, Morina N, Schick M, Watzke B, Schnyder U, Pfaltz MC. Barriers to access to outpatient mental health care for refugees and asylum seekers in Switzerland: the therapist's view. BMC Psychiatry. 2020;20(1):378.
65. APA. American Psychiatric Association - Mental Health Disparities: Diverse Populations 2017 [Available from: https://www.psychiatry.org/File%20Library/Psychiatrists/Cultural-Competency/Mental-Health-Disparities/Mental-Health-Facts-for-Diverse-Populations.pdf.
66. Lindenmeyer A, Redwood S, Griffith L, Teladia Z, Phillimore J. Experiences of primary care professionals providing healthcare to recently arrived migrants: a qualitative study. BMJ Open. 2016;6(9):e012561.
67. Kasten MJ, Berman AC, Ebright AB, Mitchell JD, Quirindongo-Cedeno O. Interpreters in Health Care: A Concise Review for Clinicians. Am J Med. 2020;133(4):424-8.e2.

68. MacFarlane A, Huschke S, Pottie K, Hauck FR, Griswold K, Harris MF. Barriers to the use of trained interpreters in consultations with refugees in four resettlement countries: a qualitative analysis using normalisation process theory. BMC Fam Pract. 2020;21(1):259.
69. Kevin P, Doug G, Mariella F, Omar E, David P, Meb R, et al. Refugees and Global Health: A Global Health E-Learning Program, Canadian Collaboration for Immigrant and Refugee Health (CCIRH) and the University of Ottawa, Canada 2013 [Available from: https://ccirhken.ca/e-learning/.
70. Jaeger FN, Pellaud N, Laville B, Klauser P. The migration-related language barrier and professional interpreter use in primary health care in Switzerland. BMC Health Serv Res. 2019;19(1):429.
71. Brisset C, Leanza Y, Rosenberg E, Vissandjée B, Kirmayer LJ, Muckle G, et al. Language barriers in mental health care: a survey of primary care practitioners. J Immigr Minor Health. 2014;16(6):1238-46.
72. GOV.UK. Vulnerable migrants: migrant health guide: Office for Health Improvement and Disparities; 2017 [Available from: https://www.gov.uk/guidance/vulnerable-migrants-migrant-health-guide.
73. GOV.UK. Office for Health Improvement and Disparities - Mental Health: Migrant Health Guide 2022 [Available from: https://www.gov.uk/guidance/mental-health-migrant-health-guide.
74. Magwood O, Kassam A, Mavedatnia D, Mendonca O, Saad A, Hasan H, et al. Mental Health Screening Approaches for Resettling Refugees and Asylum Seekers: A Scoping Review. Int J Environ Res Public Health. 2022;19(6).
75. Albritton TA, Wagner PJ. Linking cultural competency and community service: a partnership between students, faculty, and the community. Acad Med. 2002;77(7):738-9.
76. Hill L, Gray R, Stroud J, Chiripanyanga S. Inter-professional Learning to Prepare Medical and Social Work Students for Practice with Refugees and Asylum Seekers. Social Work Education. 2009;28(3):298-308.
77. Gagnon S. Interdisciplinary Rotation in Refugee Health for Undergraduate Students at Laval University with the Collaboration of a Community Organism: To Innovate to Sensibilize Physicians of Tomorrow to the Reality of Refugees just after their arrival in Canada: OP-107. Medical Education. 2011;45(1).
78. Palmer VS, Mazumder R, Spencer PS. Interprofessional global health education in a cosmopolitan community of North America: the iCHEE experience. Acad Med. 2014;89(8):1149-52.
79. von Lersner U, Ilhan KJ. Kultursensitive Psychotherapie. 1. Auflage ed: Hoegrefe Verlag; 2017.
80. Attum B, Hafiz S, Malik A, Shamoon Z. Cultural Competence in the Care of Muslim Patients and Their Families. StatPearls. Treasure Island (FL): StatPearls Publishing LLC.; 2022.
81. Rassool GH. Cultural Competence in Counseling the Muslim Patient: Implications for Mental Health. Arch Psychiatr Nurs. 2015;29(5):321-5.
82. Rourke J. Social Accountability: A Framework for Medical Schools to Improve the Health of the Populations They Serve. Acad Med. 2018;93(8):1120-4.
83. Fox AB, Earnshaw VA, Taverna EC, Vogt D. Conceptualizing and Measuring Mental Illness Stigma: The Mental Illness Stigma Framework and Critical Review of Measures. Stigma Health. 2018;3(4):348-76.
84. Stangl AL, Earnshaw VA, Logie CH, van Brakel W, C Simbayi L, Barré I, et al. The Health Stigma and Discrimination Framework: a global, crosscutting framework to inform research, intervention development, and policy on health-related stigmas. BMC Med. 2019;17(1):31.
85. Jackson-Best F, Edwards N. Stigma and intersectionality: a systematic review of systematic reviews across HIV/AIDS, mental illness, and physical disability. BMC Public Health. 2018;18(1):919.
86. Gary FA. Stigma: barrier to mental health care among ethnic minorities. Issues Ment Health Nurs. 2005;26(10):979-99.
87. Douglass CH, Lim MSC, Block K, Onsando G, Hellard M, Higgs P, et al. Exploring stigma associated with mental health conditions and alcohol and other drug use among people from migrant and ethnic minority backgrounds: a protocol for a systematic review of qualitative studies. Syst Rev. 2022;11(1):12.

88. Corneau S, Stergiopoulos V. More than being against it: anti-racism and anti-oppression in mental health services. Transcult Psychiatry. 2012;49(2):261-82.
89. Baker DW, Cameron KA, Feinglass J, Georgas P, Foster S, Pierce D, et al. Patients' attitudes toward health care providers collecting information about their race and ethnicity. J Gen Intern Med. 2005;20(10):895-900.
90. Janet H, Guerda N, Carlton G. Racism and Ethnoviolence as Trauma: Enhancing Professional and Research Training. Traumatology. 2012;18(1):65-74.
91. Drewniak D, Krones T, Wild V. Do attitudes and behavior of health care profressionals exacerbate health care disparities among immigrant and ethnic minority groups? An integrative literature review. International Journal of Nursing Studies. 2017.
92. Lee HB, Hanner JA, Cho SJ, Han HR, Kim MT. Improving access to mental health services for korean american immigrants: moving toward a community partnership between religious and mental health services. Psychiatry Investig. 2008;5(1):14-20.
93. Williams L, Gorman R, Hankerson S. Implementing a mental health ministry committee in faith-based organizations: the promoting emotional wellness and spirituality program. Soc Work Health Care. 2014;53(4):414-34.
94. WHO. Curriculum guide to support the operationalization of Refugee and Migrant Health: Global Competency Standards for Health Workers. 2021.
95. WHO. Refugee and migrant health: Global Competency Standards for health workers. 2021.
